# A Systematic Review of COVID-19 and Pericarditis

**DOI:** 10.7759/cureus.27948

**Published:** 2022-08-12

**Authors:** Pramod Theetha Kariyanna, Ahmed Sabih, Bayu Sutarjono, Kanval Shah, Alvaro Vargas Peláez, Jeremy Lewis, Rebecca Yu, Ekjot S Grewal, Apoorva Jayarangaiah, Sushruth Das, Amog Jayarangaiah

**Affiliations:** 1 Division of Interventional Cardiology, Icahn School of Medicine at Mount Sinai Morningside/Beth Israel Hospital, New York City, USA; 2 Internal Medicine, Kingsbrook Jewish Medical Center, Brooklyn, USA; 3 Emergency Medicine, Brookdale University Hospital Medical Center, New York City, USA; 4 Internal Medicine, Baystate Medical Center, Springfield, USA; 5 Internal Medicine, University of Massachusetts Medical School - Baystate Medical Center, Springfield, USA; 6 Cardiology, Mount Sinai Beth Israel, New York City, USA; 7 Internal Medicine, Saba University School of Medicine, Devens, USA; 8 Internal Medicine, New York City Health and Hospitals/Jacobi Medical Center, Bronx, USA; 9 Internal Medicine, Trinity School of Medicine, Kingstown, VCT; 10 Internal Medicine, Marshfield Clinic Health System, Marshfield, USA

**Keywords:** brain natriuretic peptide, chest x-ray, sars-cov-2, covid-19, myopericarditis, echocardiogram, pericarditis, electrocardiography (ecg), erythrocyte sedimentation rate, c-reactive protein

## Abstract

Coronavirus disease 2019 (COVID-19), caused by severe acute respiratory syndrome coronavirus 2 (SARS-CoV-2), was first identified in Wuhan, China in December 2019. Since then, the disease has spread globally, leading to the ongoing pandemic. It can cause severe respiratory illness; however, many cases of pericarditis have also been reported. This systematic review aims to recognize the clinical features of pericarditis and myopericarditis in COVID-19 patients.

Google Scholar, Medline/PubMed, CINAHL, Cochrane Central, and Web of Science databases were searched for studies reporting “Coronavirus” or “COVID” and “Peri-myocarditis,” “heart,” or “retrospective.” Case reports and retrospective studies published from May 2020 to February 2021 were reviewed.

In total, 33 studies on pericarditis, myopericarditis, and pericardial infusion were included in this review. COVID-19 pericarditis affected adult patients at any age. The incidence is more common in males, with a male-to-female ratio of 2:1. Chest pain (60%), fever (51%), and shortness of breath (51%) were the most reported symptoms, followed by cough (39%), fatigue (15%), myalgia (12%), and diarrhea (12%). Laboratory tests revealed leukocytosis with neutrophil predominance, elevated D-dimer, erythrocyte rate, and C-reactive protein. Cardiac markers including troponin-1, troponin-T, and brain natriuretic peptide were elevated in most cases. Radiographic imaging of the chest were mostly normal, and only 31% of chest X-rays showed cardiomegaly and or bilateral infiltration. Electrocardiography (ECG) demonstrated normal sinus rhythm with around 59% ST elevation and rarely PR depression or T wave inversion, while the predominant echocardiographic feature was pericardial effusion. Management with colchicine was favored in most cases, followed by non-steroidal anti-inflammatory drugs (NSAIDs), and interventional therapy was only needed when patient developed cardiac tamponade. The majority of the reviewed studies reported either recovery or no continued clinical deterioration.

The prevalence of COVID-19-related cardiac diseases is high, and pericarditis is a known extrapulmonary manifestation. However, pericardial effusion and cardiac tamponade are less prevalent and may require urgent intervention to prevent mortality. Pericarditis should be considered in patients with chest pain, ST elevation on ECG, a normal coronary angiogram, and COVID-19. We emphasize the importance of clinical examination, ECG, and echocardiogram for decision-making, and NSAIDs, colchicine, and corticosteroids are considered to be safe in the treatment of pericarditis/myopericarditis associated with COVID-19.

## Introduction and background

The novel coronavirus disease 2019 (COVID-19) is a global pandemic caused by severe acute respiratory syndrome coronavirus 2 (SARS-CoV-2) [1]. The pandemic began in Wuhan, China in December 2019. While affecting dominantly the respiratory system, COVID-19 can also cause acute and chronic damage to the cardiovascular system. The cardiovascular manifestations of COVID-19 are diverse and include arrhythmia, acute coronary syndrome, left ventricular heart failure, myocarditis, and acute and subacute pericarditis with or without pericardial effusion [2-4].

Pericarditis refers to the inflammation of the pericardium, the thin fibrous sac surrounding the heart, that can present as an isolated disease or as a manifestation of a systemic disorder, diagnosed in approximately 0.1% of patients hospitalized for chest pain. Although acute pericarditis has many causes, it is most often idiopathic or is presumed to be viral in origin [5].

Acute effusive pericarditis is a rare manifestation of COVID-19, especially without concomitant pulmonary disease or myocardial injury; yet, very little research is available regarding pericarditis caused by SARS-CoV-2. It is important to maintain a high level of suspicion to ensure early diagnosis and treatment. Diagnosing these conditions can be challenging, and early appropriate treatment can improve the outcome. Therefore, we conducted a systematic review of COVID-19 patients with acute pericarditis to assess clinical characteristics, diagnostic testing, and current treatment therapy.

Acute pericarditis and myopericarditis share the same viral etiological agents, with myocardial involvement often found in the former [6]. Therefore, we have included myopericarditis in this study.

## Review

Methodology

Protocol and Registration

The Preferred Reporting Items for Systematic Reviews and Meta-Analyses (PRISMA) checklist was followed for this systematic review [7]. The study protocol was not registered. The Joanna Briggs Institute critical appraisal tool for case reports was used in this systematic review [8].

Inclusion Criteria

Only articles reporting the association between COVID-19 and myopericarditis or pericarditis were included. Pericarditis is defined as an inflammatory process affecting the pericardial sac surrounding the heart [6]. The search terms that were used in our article screening were “COVID-19,” “Pericarditis,” “Pericardial effusion,” “Pericardial disease,” “Cardiac tamponade,” and “Myocarditis.”

Exclusion Criteria

Studies were excluded if: (1) they were not case reports, case series, or observational studies with a focus on myopericarditis or pericarditis; (2) they were reviews or editorials, (3) the patient tested negative for COVID-19; or (4) the study did not pass bias evaluation. The language of the article was not a limitation as all of the articles which were relevant in our literature search were written in the English language.

Information Sources and Search Strategies

We conducted a comprehensive literature search using Medline/PubMed, Google Scholar, CINAHL, Cochrane Central, and Web of Science databases up to and including February 28, 2021, using the terms “Coronavirus” or “COVID” and “pericarditis” or “myopericarditis.”

Study Selection

Articles were triaged based on whether titles or abstracts met the inclusion criteria. Full-text articles were then read, and those that did not satisfy the inclusion criteria were excluded. A summary of study characteristics is given in Table 1.

**Table 1:** Summary of the characteristics of included articles (n = 33). COVID-19: coronavirus disease 2019; ED: emergency department; ICU: intensive care unit

Reference, publication year	Study type	Patient profile (age in years, sex)	Symptoms	Diagnosis
Amoozgar et al., 2020 [9]	Case report	56, Male	Non-radiating exertional chest pain with dyspnea for 1 week	Acute pericarditis
Asif et al., 2020 [10]	Case report	70, Female	Chest pain, worsening dyspnea, myalgias for 3 days	Acute pericarditis
Blagojevic et al., 2020 [11]	Case report	51, Male	Sudden but persistent chest pain for 1 day; the pain was sharp, worsened with deep breathing or a change in body position, and was alleviated while sitting	Acute pericarditis
Cairns et al., 2021 [12]	Case report	58, Female	Fever, diarrhea, vomiting, poor oral intake	Acute Myopericarditis
Cizgici et al., 2020 [13]	Case report	78, Male	Chest pain and shortness of breath	Acute myopericarditis
Faraj et al., 2021 [14]	Case report	36, Male	Chest pain, worse when lying or on deep breathing	Acute pericarditis
Fox et al., 2020 [15]	Case report	43, Male	Progressive orthopnea, conversational dyspnea, and chest pain (radiating to the neck and left shoulder) for 4 days; reported mild non-productive cough and subjective fever 2 weeks prior	Acute pericarditis
Fried et al., 2020 [16]	Case series	64, Female	Persistent chest pressure for 2 days	Acute myopericarditis
García-Cruz et al., 2020 [17]	Case report	64, Male	Chest pain, dry cough, fever, dyspnea	Acute pericarditis
Inciardi et al., 2020 [18]	Case report	53, Female	Severe fatigue for 2 days, fever, and cough the week before	Acute myopericarditis
Karadeniz et al., 2020 [19]	Case report	33, Male	Retrosternal chest pain for 5 days, worse with sitting forward, unresponsive to diclofenac, severe low back pain (for 1 week)	Acute pericarditis
Kazi et al., 2020 [20]	Case report	73, Male	Dry cough, worsening fever, fatigue for 2 days before presenting to ED (6 days before transfer to ICU); dyspnea developed over next 4 days	Acute myopericarditis
Khalid et al., 2020 [21]	Case series	34, Female	Chest heaviness, generalized weakness, subjective fever/chills, body aches for 3 days	Acute myopericarditis
Khatri et al., 2020 [22]	Case report	50, Male	Fever, chills, generalized malaise, non-productive cough, dyspnea for 3-4 days, and an episode of near-syncope on the day of presentation	Acute myopericarditis
Kumar et al., 2020 [23]	Case report	66, Male	Acute-onset severe pleuritic chest pain for 1 day (4 episodes lasting 10-15 minutes); pain worse lying down, relieved by leaning forward	Acute pericarditis
Legrand et al., 2020 [24]	Case report	39, Male	Chest pain and dyspnea for 2 days	Acute myopericarditis
Li et al., 2020 [25]	Case report	60, Male	fever, cough, worsening dyspnea, mild abdominal pain, diarrhea for 8 days	Acute myopericarditis
Marschall et al., 2020 [26]	Case report	45, Male	Dyspnea with minimal exertion, orthopnea, bendopnea	Acute pericarditis
Naqvi et al., 2020 [27]	Case report	55, Male	Chest pain for 24 hours	Acute pericarditis
Ortiz-Martínez et al., 2020 [28]	Case report	25, Male	Myalgias, arthralgias, diarrhea, 2 days later: fever and nausea, began isolation, on 8th day: intense pleuritic centrothoracic chest pain, improved with sitting forward, worse with supine, dyspnea at rest	acute pericarditis
Özturan et al., 2020 [29]	Case report	25, Male	Acute onset chest pain and shortness of breath, 4-day history of progressive fatigue and fever	Acute myopericarditis
Patel et al, 2021 [30]	Case report	63, Male	Fever, dry cough, and malaise for 1 week; chest pain for 1 day	Acute pericarditis
Purohit et al., 2020 [31]	Case report	82, Female	Productive cough, fever with chills, intermittent diarrhea for 5 days	Acute myopericarditis
Raymond et al., 2020 [32]	Case report	7, Female	Cough, chest pain, orthopnea for 3 days	Acute pericarditis
Recalcati et al., 2020 [33]	Case report	19, Female	Fever followed by chest pain, cutaneous rash	Acute myopericarditis
Sampaio et al., 2020 [34]	Case report	45, Female	Dyspnea, fever, myalgia, postural hypotension progressing over 7 days	Acute myopericarditis
Sandino Pérez et al., 2020 [35]	Case report	73, Male	Cough, dyspnea on moderate exertion, fever for 4 days	Acute pericarditis
Sauer et al., 2020 [36]	Case series	60, Male	Asthenia for 1 week, acute anosmia, one month later prescribed amoxicillin-clavulanic acid for shivers and developed diffuse erythema 2 days later	Acute myopericarditis
Shah et al., 2020 [37]	Case series	38, Male	Hospitalized for 3 weeks with COVID-19 pneumonia, presented to ED a few hours after discharge with pricking chest pain not related to breathing, exertion, or posture	Acute myopericarditis
Thrupthi et al., 2021 [38]	Case report	68, Male	Chest tightness for 5 days, dry cough, mild fatigue, shortness of breath on exertion, chest pain	Acute pericarditis
Tiwary et al., 2020 [39]	Case report	30, Male	Bilateral abdominal flank pain, shortness of breath, fatigue, tiredness, lightheadedness	Myopericarditis
Tung-Chen et al., 2020 [40]	Case report	35, Female	Dry cough, anosmia, malaise, low-grade fever	Acute pericarditis
Walker et al., 2020 [41]	Case report	30, Female	Fever, dry cough, exertional dyspnea for 3 days	Acute pericarditis

Data Collection Process and Data Items

Data extracted from articles included the name of the first author, year of publication, country, and study design. Variables for which data were sought from all studies included patient age and sex and presenting complaints at the time of admission. Laboratory tests, diagnostic studies, management of pericarditis, and patient outcomes including complications were extrapolated from case studies.

Analysis of Results and Summary of Measures

Information was reviewed if it was reported by two or more articles. Subsequently, the data were tabulated, evaluated, and summarized.

Risk of Bias Across Studies

Potential bias across studies was analyzed within study characteristics. Two independent reviewers evaluated the methodological quality of the eligible studies. A third reviewer evaluated papers when there was no agreement. The Joanna Briggs Institute critical appraisal tool for case reports was used in this systematic review [8]. Bias was evaluated using a checklist of eight questions. Each question is specified in the Appendix concerning the risk of bias whereby an overall appraisal was made of each article: low risk of bias (included), high risk of bias (excluded), or uncertain risk of bias (more information is required). For this study, an answer of “yes” equal to or greater than 50% of the questions was considered to be low risk of bias. Similarly, an answer of “no” equal to or greater than 50% of questions was determined to be high risk of bias, whereas “unclear” answers were equal to or greater than a 50% response.

Results

Study Selection

**Figure 1:**
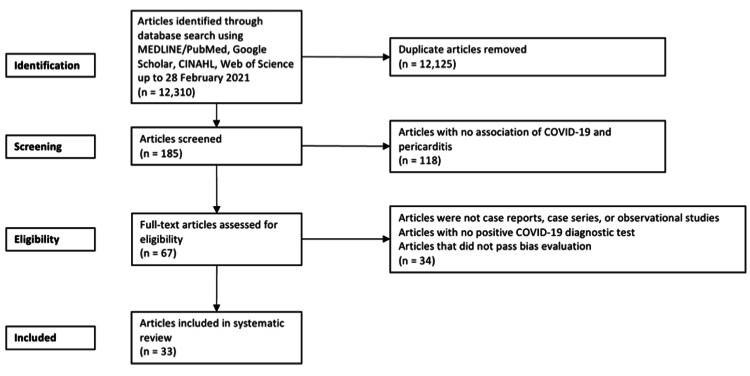
Flow diagram of the literature search and selection criteria adapted from the Preferred Reporting Items for Systematic Reviews and Meta-Analyses (PRISMA).

From the five databases, 12,310 articles were selected concerning COVID-19 and myocarditis. In total, 34 articles were selected once assessed for eligibility [9-42]. The study by Rauch et al. [42] was removed from this list as it did not meet the minimum criteria required when assessed for bias. A PRISMA flow diagram detailing the process of identification, inclusion, and exclusion of studies is shown in Figure 1.

Study Characteristics

In total, 29 articles selected for this study were case reports [9-15,17-20,22-35,38-41] while four were case series [16,21,36,37]. All articles were published in 2020 [9-11,13,15-29,31-37,39-41], except four, which were published in 2021 [12,14,30,38]. The majority of the studies were conducted in the United States [9,10,15,16,20-22,25,30-32,38-41], followed by countries from the European Union or United kingdom [11,12,18,23,24,26,33,35,36,40] and the Middle East or Asia [12,19,27,29,37]. Only three studies originated from Latin America [17,28,34] and one from northern Africa [14].

Risk of Bias Within Studies

In comparison to case reports, the majority of articles were rated to have a low risk of bias [9-41]. As mentioned previously, only one study was characterized as high risk of bias [42] and was removed from the systematic review.

Results of Individual Studies

The common theme of the studies was either the identification of only pericarditis [10,11,14,16,19,23,26,28,30,35,37,38,40], myopericarditis [13,18,20,21,24,25,29,33,34], or the association of cardiac tamponade or pericardial effusion and either pericarditis or myopericarditis [9,12,15,17,22,27,31,32,36,39,41] in COVID-19 patients.

Patient Profile

COVID-19 patients selected for the systematic review were 49.3 ± 18.5 years of age, with more males affected than females at a ratio of 2:1 [9-41]. The eldest was 82 years old [31], while the youngest was seven years old [32].

Presenting Complaints

The predominant symptom at admission was chest pain [9,11,13-17,19,21,23,24,27-30,32,33,37,38], followed by fever [12,15,17,18,20-22,25,28-31,33-35,40,41], shortness of breath [10,13,15,17,20,22,24-26,28,29,32,34,35,38,39,41], and cough [15,17,18,20,22,25,30-32,35,38,40,41]. The mean temperature recorded was 37.5 ± 1.1°C [9-12,14,16-21,22,30,32,33,35-38,41] while the mean blood pressure was 112.0 ± 20.3/71.7 ± 16.0 [3-12,14-21,23,26-30,34,37,38,41]. The average heart rate was also elevated at 103.9 ± 21.3 beats/minute [9-12,14-21,23-30,32,34,37,38,41]. The mean oxygen saturation was 96.2 ± 0.1% [9-12,14-21,23,25-30,33,34,36-38,41]. The distribution of presenting complaints and associated symptoms are listed in Table 2.

**Table 2:** Most common clinical manifestations on admission of patients with COVID-19 and pericarditis and myopericarditis as the proportion reported from all articles (n = 33).

Symptom	%
Chest pain	60.6
Fever	51.5
Shortness of breath	51.5
Cough	39.4

Medical History

Nearly half of the patients reported a medical history of hypertension [10-13,16,20,22,23,25,27,30,31,38,39], followed by diabetes mellitus [10,12,20,37,39] and hyperlipidemia [10,16,25,31].

Laboratory Tests

A summary of laboratory tests is presented in Table 3.

**Table 3:** Trends of laboratory values of COVID-19 patients with pericarditis and myopericarditis from all articles (n = 33). SaO_2_: oxygen saturation (arterial blood); WBC: white blood cell; CRP: C-reactive protein; ESR: erythrocyte sedimentation rate; CK-MB: creatine kinase-myoglobin binding; BNP: brain natriuretic peptide; COVID-19: coronavirus disease 2019

	Trends	(Standard range)
Vitals		
Temperature, °C (n = 25)	Elevated	(<37.5)
Heart rate, beats/minute (n = 25)	Elevated	
Systolic blood pressure, mmHg (n = 22)	Normal	(90–120)
Diastolic blood pressure, mmHg (n = 21)	Normal	(60–80)
SaO_2_, % (n = 25)	Normal	(>94)
Inflammatory markers		
WBC, cells/mm^3^ (n = 17)	Elevated	(4,500–11,000)
WBC predominance (n = 8)	Neutrophils	
CRP, mg/L (n = 21)	Elevated	(<8.0)
ESR, mm/hour (n = 5)	Elevated	(0–20)
D-dimer level, ng/mL (n = 15)	Elevated	(<250)
Cardiac markers		
Troponin-I, ng/mL (n = 17)	Elevated	<0.04
Troponin-T, ng/mL (n = 13)	Elevated	<0.01
CK-MB, ng/mL (n = 3)	Elevated	<5.0
BNP, pg/mL (n = 10)	Elevated	<125

All patients were confirmed COVID-19 positive [9-41]. The majority of studies reported leukocytosis [9,11,14,15,20,22,24-29,32-35,37,40] and neutrophil predominance [11,20,21,25,34,38-40], with a mean of 15,622 ± 2,654 cells/mm3. Increased C-reactive protein (CRP) was also reported by the majority of studies, with a mean of 15.0 ± 9.59 mg/dL [11,13-16,19-22,24-26,28-30,32-35,37-40]. Only six studies recorded erythrocyte sedimentation rate (ESR), with three reporting elevated values at a mean of 59.3 ± 25.7 mm/hour [11,21,22,30,32,38].

Two-thirds of studies showed elevated D-dimer levels [9,15,16,19,22,23,25,26,28,33-35,38,40,41], with two articles reporting values higher than 5,000 ng/mL [9,15]. Only 60.0% of studies reported either an increased troponin-I [9,11,15,16,19,21,22,24,27,29,31,32,34,36,38-40] or troponin-T [12-14,18,20,23,25,26,28,30,33,35,37]. The troponin-I values ranged from 0.544 ng/mL to 90.0 ng/mL, while mean troponin-T values ranged from 0.367 to 13.0 ng/mL. Nine out of 10 studies recorded elevated n-terminal brain natriuretic peptide (BNP), with a mean value of 4,770.1 ± 5,158.2 pg/mL [11,18,21,24,25,32,34,38,39,41]. Only three studies reported elevated creatine kinase-MB results, with a mean of 39.9 ± 13.3 ng/mL [22,25,29].

Diagnostic Studies

A summary of diagnostic studies is provided in Table 4.

**Table 4:** Common findings found on diagnostic tests of COVID-19 patients with pericarditis and myopericarditis as the proportion recorded from all articles (n = 33). LVEF: left ventricular ejection fraction; CT: computed tomography; MRI: magnetic resonance imaging; COVID-19: coronavirus disease 2019

Diagnostic study	%
Electrocardiography (n = 32)	
ST-segment elevation	59
PR depression	28
Tachycardia	22
T-wave inversion	13
Electric alternans	3
Echocardiography (n = 30)	
Pericardial effusion	67
Hypokinesis	20
Reduced LVEF	20
Ventricular wall thickening	13
Imaging (X-ray) (n = 16)	
Bilateral infiltrates	31
Cardiomegaly	31
Imaging (CT) (n = 18)	
Ground-glass opacities	50
Pericardial effusion	39
Pleural effusion	33
Imaging (MRI) (n = 4)	
Late gadolinium enhancements	50

The predominant feature of electrocardiogram (ECG) recordings was sinus rhythm [10-11,15,16,20-22,32,33,36,40,41], with ST-segment elevation [10-11,13,15-18,20,22-27,30,33,35,37,38]. About one in four articles reported PR depression [10,11,15,20,23,26,29,30,37] while one in five presented with tachycardia [15,16,21,22,32,33,41]. Finally, inverted T waves were found in one in 10 studies [10,31,32,40] while only one study had the classic presentation of electric alternans [32].

Echocardiography showed pericardial infusion in nearly three out of four studies [9-12,14,15,17-19,21-22,24,25,27,28,30-32,34,35,39,41] while one in four demonstrated either hypokinesis [16,18,20,25,29,31] or reduced left ventricular ejection fraction (<50%) [16-18,21,25,29].

Less than one-third of the articles reported bilateral infiltrates on chest X-ray [10,17,26,37,38], with the same proportion highlighting cardiomegaly [9,10,14,15,32]. Likewise, computed tomography (CT) imaging of the chest revealed similar results, with 50% of images demonstrating ground-glass opacities in the lungs [9,13,19,24,28,34,39] while one in three reporting pleural effusion [9,14,19,24,34,39]. Pericardial effusion was reported in slightly more than one-third of articles [9,13,19,24,28,34,39]. Magnetic resonance imaging (MRI) was the least used modality, with only half demonstrating subepicardial delayed gadolinium enhancement [24,36].

Management of Myocarditis

The most common medical management was the use of colchicine by 45.1% of studies [14,15,19,21,23,24,26,28,30,32,35-38,40,41], followed by either aspirin or non-steroidal anti-inflammatory drugs (NSAIDs) in 41.9% of articles [11,15,19,26-30,32,33,37,38,41] and corticosteroids in 35.3% of patients [18,21,22,25,28,34,35,38]. Regarding surgical interventions, pericardiocentesis [10,12,15,21,22,27,31,32,34] or the creation of pericardial windows [10,17,39,41] were required in 13 case reports.

Outcomes

The outcomes of the patients were documented in the majority of case reports. Only two deaths were reported [20,22], with the majority being discharged from the hospital [9-19,21,23-41].

Risk of Bias Across Studies

Due to the nature of descriptive studies, the results being presented are liable to the investigator, procedure, and selection bias.

Limitation of the Study

Statistical analyses were not performed as there were no control/comparison groups in the included studies.

Discussion

Pathophysiology

SARS-CoV-2 is thought to lead to myocardial damage and inflammation through the following processes: (1) direct invasion, (2) ischemic injury, and (3) cytokine storm. SARS-CoV-2 typically invades the respiratory epithelium through direct invasion via binding to angiotensin-converting enzyme-2 (ACE2) receptors on the host cells. These receptors are predominantly found on the respiratory epithelium and are also expressed on cardiac cells, facilitating a pathway and allowing for direct invasion and damage to cardiomyocytes [43]. Hypoxemia, which can result from pulmonary compromise from SARS-CoV-2 infection, can also lead to impaired myocardial oxygen supply resulting in a supply and demand mismatch, which then leads to ischemic injury to cardiomyocytes [43]. Cytokine storm is a known physiologic mechanism associated with SARS-CoV-2 infection, leading to the widespread production of high levels of pro-inflammatory cytokines. Large-scale production of these pro-inflammatory cytokines has also been linked to myocardial damage through recorded elevations in troponin levels during these states [43]. Autopsy reports on patients who died of COVID-19 infection have also shown findings within cardiac tissue consistent with inflammatory cellular infiltrate and multi-nucleated giant cells. Additionally, findings of pericardial effusions have also been seen in autopsy examinations [43].

Clinical Presentation

The clinical manifestations of COVID-19 in order of prevalence are fever, cough, and fatigue [2]. A common clinical manifestation of pericarditis is typically sharp and pleuritic chest pain that radiates posteriorly to the bilateral trapezius ridges, which improves on sitting up or leaning forward [44]. The presence of fever, subacute course, large effusion, or tamponade are indicators of poor prognosis [45]. Auscultation of the left sternal border typically indicates a triphasic pericardial friction rub. Here, we identified that patients with COVID-19 pericarditis commonly presented with chest pain, in addition to fever and shortness of breath.

Laboratory Findings

It has been shown that thrombo-inflammatory biomarkers are associated with COVID-19 infection severity [46]. Our systematic review showed that COVID-19 patients with pericarditis exhibited leukocytosis with neutrophil predominance, elevated inflammatory markers (ESR and CRP), and elevated D-dimer levers as well. These case studies also exhibited elevated cardiac markers (troponin-I, troponin-T, CK-MB, and BNP) as well. Typically, 32-50% of cases of viral and idiopathic acute pericarditis as evidence of inflammatory myocardial damage and possible evidence of myopericarditis [47]. Patients with pericarditis and concomitant myocarditis have a potential for higher risk of complications, including left ventricular dysfunction and possible heart failure [48]. Similarly, our review showed that the deaths were from COVID-19 patients diagnosed with myopericarditis. Similarly, of the 10 studies to report COVID-19-related complications, eight of which were present in COVID-19 patients diagnosed with myopericarditis [20-22,24,25,34,36,39]. Furthermore, the only two deaths that were reported were from patients diagnosed with myopericarditis [20,22].

Imaging

The typical ECG presentation of a COVID-19 patient with pericarditis is sinus rhythm with ST-segment elevation, while a minority of individuals exhibit PR-segment depression or tachycardia. ECG presentation of widespread ST-segment elevation and PR depression, although this finding is considered characteristic for pericarditis, es only found in slightly more than half of the patients with acute pericarditis, particularly among younger males [44]. Disease progression may evolve to include T waves [44].

Echocardiographic patterns of the brightness of the pericardial layers, which are associated with fibrinous pericarditis, and pericardial effusion have been described in pericarditis [44]. Approximately 60% of patients with acute pericarditis were found to have developed a pericardial effusion, although its absence does not exclude pericarditis. Our systematic review found that the predominant feature found in the majority of patients with pericarditis is pleural effusion, while a minority of patients have hypokinesis or depressed ejection fraction.

Chest radiograph findings in patients with acute pericarditis are usually normal except in the setting of very large pericardial effusion, which would convey the characteristic water bottle-shaped cardiac silhouette [44]. CT and cardiac MRI with gadolinium contrast are recommended as secondary imaging techniques after echocardiography and chest radiographs to detect and confirm pericardial inflammation, assess pericardial thickening or late gadolinium enhancement, and identify the presence of effusion or constrictive physiology [44,49]. Likewise, the case resorts in our systematic reported predominantly COVID-19 disease of the lungs with bilateral infiltrates or ground-glass opacities, with characteristic pericarditis features of pericardial effusion or cardiomegaly demonstrated in a minority of patients. In diagnostic CT scans, findings of pericardial effusions were also studied and reported to be present in a small number of patients as well, and the degree of pericardial effusion was also seen to correlate to the severity of infection. Evidence of myocarditis was also reported on CT scans, with increases in cardiac wall thickness, myocardial edema, and wall hypokinesia. In cardiac MRIs, these studies are limited; however, there have been reports of myocardial edema and significant findings of wall motion abnormalities in patients already clinically diagnosed with acute myocarditis. The use of positron emission tomography scan in detailing diagnostic findings of pericardial effusions and cardiomegaly only highlighted small studies which did not reveal any evidence of cardiac involvement [50].

Treatment

There was no prevailing strategy regarding the medical management to treat pericarditis in patients with COVID-19. We found that colchicine, aspirin, NSAIDs, and/or corticosteroids were used in the majority of studies. NSAIDs, most commonly ibuprofen (600 to 800 mg every six to eight hours), indomethacin (25 to 50 mg every eight hours), and aspirin (2 to 4 g daily in divided doses) have long been the mainstay of medical therapy for both acute and recurrent pericarditis whether or not it is of idiopathic or viral causes [41,51-53]. Corticosteroid therapy (0.2 to 0.5 mg of prednisone per kg of body weight daily) used to be the initial choice in treating pericarditis with pericardial effusions or recurrent episodes unresponsive to aspirin or NSAIDs; however, it has been shown to be associated with an increased risk in adverse effects and higher recurrence risk in non-randomized studies [44,52]. There is considerable evidence highlighting that the use of colchicine is efficacious and safe for the treatment and prevention of both acute and recurrent pericarditis, as well as for reducing rehospitalizations and symptom duration in the process [54-58]. In the cases that were reviewed in our study, the majority utilized colchicine (17/33), and 13 cases utilized NSAIDs either in the form of aspirin, ibuprofen, or indomethacin, all of which were usually used in conjunction with colchicine, with the exception of 5/13 of the cases. Among 10/39 of the cases, there was no use of NSAID or colchicine therapy. Additionally, one case reported the use of Anakinra [19] after noting poor results with NSAID and colchicine therapy. All patients received some form of standardized COVID-19 therapy as well, including intravenous corticosteroids. All of the doses reported in the treatment of myopericarditis and pericarditis were within the appropriate dosages discussed in the standard therapies above.

## Conclusions

Our systematic review provides a comprehensive characterization of the clinical features among COVID-19 patients with pericarditis. Currently, as data are limited, more research is needed to improve our understanding of COVID-19 pericarditis.

## Appendices

**Table 5:** Risk of bias across studies.

Reference, publication year	Were patient’s demographic characteristics clearly described?	Was the patient’s history clearly described and presented as a timeline?	Was the current clinical condition of the patient on presentation clearly described?	Were diagnostic tests or assessment methods and results clearly described?	Was the intervention(s) or treatment procedure(s) clearly described?	Was the post-intervention clinical condition clearly described?	Were adverse events (harms) or unanticipated events identified and described?	Does the case report provide takeaway lessons?	Total score
Amoozgar et al., 2020 [9]	Some information missing	√	√	√	√	√	√	√	87.5%
Asif et al., 2020 [10]	√	√	√	Some information missing	Some information missing	√	√	√	75.0%
Blagojevic et al., 2020 [11]	√	√	√	√	√	√	√	√	100.0%
Cairns et al., 2021 [12]	√	√	√	Some information missing	√	√	√	√	87.5%
Cizgici et al., 2020 [13]	Some information missing	√	Some information missing	√	All information missing	All information missing	√	√	50.0%
Faraj et al., 2021 [14]	Some information missing	√	√	√	√	√	√	√	87.5%
Fox et al., 2020 [15]	√	√	Some information missing	√	√	√	√	√	87.5%
Fried et al., 2020 [16]	Some information missing	√	√	All information missing	√	√	√	√	75.0%
García-Cruz et al., 2020 [17]	√	All information missing	√	Some information missing	All information missing	All information missing	√	√	50.0%
Inciardi et al., 2020 [18]	√	√	√	√	√	√	√	√	100.0%
Karadeniz et al., 2020 [19]	Some information missing	√	√	√	√	√	√	√	87.5%
Kazi et al., 2020 [20]	√	√	√	√	Some information missing	√	√	√	87.5%
Khalid et al., 2020 [21]	Some information missing	√	√	√	Some information missing	√	√	√	75.0%
Khatri et al., 2020 [22]	√	√	Some information missing	√	Some information missing	√	√	√	75.0%
Kumar et al., 2020 [23]	Some information missing	√	√	√	Some information missing	√	√	√	75.0%
Legrand et al., 2020 [24]	Some information missing	√	Some information missing	√	√	√	√	√	75.0%
Li et al., 2020 [25]	Some information missing	√	√	√	√	√	√	√	87.5%
Marschall et al., 2020 [26]	Some information missing	√	√	√	Some information missing	√	√	√	75.0%
Naqvi et al., 2020 [27]	√	√	√	Some information missing	√	All information missing	All information missing	√	62.5%
Ortiz-Martinez et al., 2020 [28]	Some information missing	√	√	√	√	√	√	√	87.5%
Özturan et al., 2020 [29]	Some information missing	√	√	√	Some information missing	√	√	√	75.0%
Patel et al, 2021 [30]	Some information missing	√	√	√	√	√	√	√	87.5%
Purohit et al., 2020 [31]	Some information missing	√	√	√	√	√	√	√	87.5%
Raymond et al., 2020 [32]	Some information missing	√	√	√	Some information missing	√	√	√	75.0%
Recalcati et al., 2020 [33]	Some information missing	√	√	√	Some information missing	All information missing	√	√	62.5%
Sampaio et al., 2020 [34]	Some information missing	√	Some information missing	√	√	√	√	√	75.0%
Sandino Pérezet al., 2020 [35]	Some information missing	√	√	All information missing	√	√	√	√	75.0%
Sauer et al., 2020 [36]	Some information missing	√	Some information missing	Some information missing	√	√	√	√	62.5%
Shah et al., 2020 [37]	Some information missing	√	√	√	Some information missing	Some information missing	Some information missing	√	50.0%
Thrupthi et al., 2021 [38]	Some information missing	√	√	Some information missing	√	√	√	√	75.0%
Tiwary et al., 2020 [39]	√	√	Some information missing	√	Some information missing	√	√	√	75.0%
Tung-Chen et al., 2020 [40]	Some information missing	√	Some information missing	√	√	√	√	√	75.0%
Walker et al., 2020 [41]	√	√	√	√	Some information missing	√	√	√	75.0%
Rauch et al., 2020 [42]	No	All information missing	All information missing	All information missing	All information missing	All information missing	√	√	25.0%

## References

[1] (2022). Report of the WHO-China Joint Mission on Coronavirus Disease 2019 (COVID-19). https://www.who.int/docs/default-source/coronaviruse/who-china-joint-mission-on-covid-19-final-report.pdf.

[2] Fu L, Wang B, Yuan T (2020). Clinical characteristics of coronavirus disease 2019 (COVID-19) in China: a systematic review and meta-analysis. J Infect.

[3] Zheng YY, Ma YT, Zhang JY, Xie X (2020). COVID-19 and the cardiovascular system. Nat Rev Cardiol.

[4] Otaal PS, Batta A, Makkar K, Vijayvergiya R (2020). Cardiovascular conundrums of COVID-19 pandemic. J Postgrad Med Educ Res.

[5] Adler Y, Charron P, Imazio M (2015). 2015 ESC Guidelines for the diagnosis and management of pericardial diseases: The Task Force for the Diagnosis and Management of Pericardial Diseases of the European Society of Cardiology (ESC) Endorsed by: The European Association for Cardio-Thoracic Surgery (EACTS). Eur Heart J.

[6] Imazio M, Cecchi E, Demichelis B (2008). Myopericarditis versus viral or idiopathic acute pericarditis. Heart.

[7] Moher D, Shamseer L, Clarke M (2015). Preferred reporting items for systematic review and meta-analysis protocols (PRISMA-P) 2015 statement. Syst Rev.

[8] Moola S, Munn Z, Sears K (2015). Conducting systematic reviews of association (etiology): the Joanna Briggs Institute's approach. Int J Evid Based Healthc.

[9] Amoozgar B, Kaushal V, Mubashar U, Sen S, Yousaf S, Yotsuya M (2020). Symptomatic pericardial effusion in the setting of asymptomatic COVID-19 infection: a case report. Medicine (Baltimore).

[10] Asif T, Kassab K, Iskander F, Alyousef T (2020). Acute pericarditis and cardiac tamponade in a patient with COVID-19: a therapeutic challenge. Eur J Case Rep Intern Med.

[11] Blagojevic NR, Bosnjakovic D, Vukomanovic V, Arsenovic S, Lazic JS, Tadic M (2020). Acute pericarditis and severe acute respiratory syndrome coronavirus 2: case report. Int J Infect Dis.

[12] Cairns L, Abed El Khaleq Y, Storrar W, Scheuermann-Freestone M (2021). COVID-19 myopericarditis with cardiac tamponade in the absence of respiratory symptoms: a case report. J Med Case Rep.

[13] Cizgici AY, Zencirkiran Agus H, Yildiz M (2020). COVID-19 myopericarditis: it should be kept in mind in today's conditions. Am J Emerg Med.

[14] Faraj R, Belkhayat C, Bouchlarhem A, El Aidouni G, Bkiyar H, Housni B (2021). Acute pericarditis revealing COVID-19 infection: case report. Ann Med Surg (Lond).

[15] Fox K, Prokup JA, Butson K, Jordan K (2020). Acute effusive pericarditis: a late complication of COVID-19. Cureus.

[16] Fried JA, Ramasubbu K, Bhatt R (2020). The variety of cardiovascular presentations of COVID-19. Circulation.

[17] García-Cruz E, Manzur-Sandoval D, Lazcano-Díaz EA, Soria-Castro E, Jiménez-Becerra S (2020). Cardiac tamponade in a patient with myocardial infarction and COVID-19: electron microscopy. JACC Case Rep.

[18] Inciardi RM, Lupi L, Zaccone G (2020). Cardiac involvement in a patient with coronavirus disease 2019 (COVID-19). JAMA Cardiol.

[19] Karadeniz H, Yamak BA, Özger HS, Sezenöz B, Tufan A, Emmi G (2020). Anakinra for the treatment of COVID-19-associated pericarditis: a case report. Cardiovasc Drugs Ther.

[20] Kazi DS, Martin LM, Litmanovich D, Pinto DS, Clerkin KJ, Zimetbaum PJ, Dudzinski DM (2020). Case 18-2020: a 73-year-old man with hypoxemic respiratory failure and cardiac dysfunction. N Engl J Med.

[21] Khalid N, Chen Y, Case BC (2020). COVID-19 (SARS-CoV-2) and the heart - an ominous association. Cardiovasc Revasc Med.

[22] Khatri A, Wallach F (2020). Coronavirus disease 2019 (Covid-19) presenting as purulent fulminant myopericarditis and cardiac tamponade: a case report and literature review. Heart Lung.

[23] Kumar R, Kumar J, Daly C, Edroos SA (2020). Acute pericarditis as a primary presentation of COVID-19. BMJ Case Rep.

[24] Legrand F, Chong-Nguyen C, Ghanem N (2020). Myopericarditis, rhabdomyolysis, and acute hepatic injury: sole expression of a SARS-CoV-2 infection. Circ Cardiovasc Imaging.

[25] Li A, Garcia-Bengochea Y, Stechel R, Azari BM (2020). Management of COVID-19 myopericarditis with reversal of cardiac dysfunction after blunting of cytokine storm: a case report. Eur Heart J Case Rep.

[26] Marschall A, Concepción Suárez R, Dejuan Bitriá C, Fernández Pascual MC (2020). Acute pericarditis secondary to COVID-19. Emergencias.

[27] Naqvi SG, Naseeb U, Fatima K, Riffat S, Memon AG (2020). Acute pericarditis and pericardial effusion in a hypertensive COVID-19 patient. Cureus.

[28] Ortiz-Martínez Y, Cabeza-Ruiz LD, Vásquez-Lozano SH, Villamil-Gómez WE, Rodriguez-Morales AJ (2020). Pericarditis in a young internal medicine resident with COVID-19 in Colombia. Travel Med Infect Dis.

[29] Özturan İU, Köse B, Özkan B, Köse A (2020). Myopericarditis caused by severe acute respiratory syndrome coronavirus 2. Clin Exp Emerg Med.

[30] Patel VD, Patel KH, Lakhani DA, Desai R, Mehta D, Mody P, Pruthi S (2021). Acute pericarditis in a patient with severe acute respiratory syndrome coronavirus 2 (SARS-CoV-2) infection: a case report and review of the literature on SARS-CoV-2 cardiological manifestations. AME Case Rep.

[31] Purohit R, Kanwal A, Pandit A (2020). Acute myopericarditis with pericardial effusion and cardiac tamponade in a patient with COVID-19. Am J Case Rep.

[32] Raymond TT, Das A, Manzuri S, Ehrett S, Guleserian K, Brenes J (2020). Pediatric COVID-19 and pericarditis presenting with acute pericardial tamponade. World J Pediatr Congenit Heart Surg.

[33] Recalcati S, Piconi S, Franzetti M, Barbagallo T, Prestinari F, Fantini F (2020). Colchicin treatment of COVID-19 presenting with cutaneous rash and myopericarditis. Dermatol Ther.

[34] Sampaio PP, Ferreira RM, de Albuquerque FN (2021). Rescue venoarterial extracorporeal membrane oxygenation after cardiac arrest in COVID-19 myopericarditis: a case report. Cardiovasc Revasc Med.

[35] Sandino Pérez J, Aubert Girbal L, Caravaca-Fontán F, Polanco N, Sevillano Prieto Á, Andrés A (2020). Pericarditis secundaria a infección por COVID-19 en un paciente trasplantado renal. Nefrologia.

[36] Sauer F, Dagrenat C, Couppie P, Jochum G, Leddet P (2020). Pericardial effusion in patients with COVID-19: case series. Eur Heart J Case Rep.

[37] Shah JZ, Kumar SA, Patel AA (2020). Myocarditis and pericarditis in patients with COVID-19. Heart Views.

[38] Thrupthi K, Ganti A, Acherjee T, Mehmood MA, Vakde T (2021). A rare case of acute pericarditis due to SARS-CoV-2 managed with aspirin and colchicine. Cureus.

[39] Tiwary T, Baiswar S, Jinnur P (2020). A rare case of COVID-19 myocarditis with cardiac tamponade in a young diabetic adult with renal failure. Cureus.

[40] Tung-Chen Y (2020). Acute pericarditis due to COVID-19 infection: an underdiagnosed disease?. Med Clin (Engl Ed).

[41] Walker C, Peyko V, Farrell C, Awad-Spirtos J, Adamo M, Scrocco J (2020). Pericardial effusion and cardiac tamponade requiring pericardial window in an otherwise healthy 30-year-old patient with COVID-19: a case report. J Med Case Rep.

[42] Rauch S, Regli IB, Clara A, Seraglio PM, Bock M, Poschenrieder F, Resch M (2020). Right ventricular myopericarditis in COVID-19: a call for regular echocardiography. Minerva Anestesiol.

[43] Geng YJ, Wei ZY, Qian HY, Huang J, Lodato R, Castriotta RJ (2020). Pathophysiological characteristics and therapeutic approaches for pulmonary injury and cardiovascular complications of coronavirus disease 2019. Cardiovasc Pathol.

[44] Imazio M, Gaita F, LeWinter M (2015). Evaluation and treatment of pericarditis: a systematic review. JAMA.

[45] Imazio M, Cecchi E, Demichelis B (2007). Indicators of poor prognosis of acute pericarditis. Circulation.

[46] Chaudhary R, Garg J, Houghton DE (2021). Thromboinflammatory biomarkers in COVID-19: systematic review and meta-analysis of 17,052 patients. Mayo Clin Proc Innov Qual Outcomes.

[47] Imazio M, Brucato A, Cumetti D (2008). Corticosteroids for recurrent pericarditis: high versus low doses: a nonrandomized observation. Circulation.

[48] Imazio M, Brucato A, Spodick DH, Adler Y (2014). Prognosis of myopericarditis as determined from previously published reports. J Cardiovasc Med (Hagerstown).

[49] Ardhanari S, Yarlagadda B, Parikh V, Dellsperger KC, Chockalingam A, Balla S, Kumar S (2017). Systematic review of non-invasive cardiovascular imaging in the diagnosis of constrictive pericarditis. Indian Heart J.

[50] Mishra AK, Lal A, Sahu KK, Kranis M, Sargent J (2020). Quantifying and reporting cardiac findings in imaging of COVID-19 patients. Monaldi Arch Chest Dis.

[51] LeWinter MM (2014). Clinical practice. Acute pericarditis. N Engl J Med.

[52] Sheth S, Wang DD, Kasapis C (2010). Current and emerging strategies for the treatment of acute pericarditis: a systematic review. J Inflamm Res.

[53] Horneffer PJ, Miller RH, Pearson TA, Rykiel MF, Reitz BA, Gardner TJ (1990). The effective treatment of postpericardiotomy syndrome after cardiac operations. A randomized placebo-controlled trial. J Thorac Cardiovasc Surg.

[54] Siak J, Flint N, Shmueli HG, Siegel RJ, Rader F (2021). The use of colchicine in cardiovascular diseases: a systematic review. Am J Med.

[55] Lutschinger LL, Rigopoulos AG, Schlattmann P, Matiakis M, Sedding D, Schulze PC, Noutsias M (2019). Meta-analysis for the value of colchicine for the therapy of pericarditis and of postpericardiotomy syndrome. BMC Cardiovasc Disord.

[56] Verma S, Eikelboom JW, Nidorf SM, Al-Omran M, Gupta N, Teoh H, Friedrich JO (2015). Colchicine in cardiac disease: a systematic review and meta-analysis of randomized controlled trials. BMC Cardiovasc Disord.

[57] Imazio M, Brucato A, Cemin R (2013). A randomized trial of colchicine for acute pericarditis. N Engl J Med.

[58] Imazio M, Bobbio M, Cecchi E (2005). Colchicine in addition to conventional therapy for acute pericarditis: results of the COlchicine for acute PEricarditis (COPE) trial. Circulation.

